# Overcoming Ethical Challenges in Conducting Research Among Persons With Dementia

**DOI:** 10.1093/geroni/igaa057.066

**Published:** 2020-12-16

**Authors:** Jeanine Yonashiro Cho, Elizabeth Avent, Roberta Peterson, Laura Mosqueda, Zachary Gassoumis

**Affiliations:** 1 Keck School of Medicine of USC, Los Angeles, California, United States; 2 University of Southern California, Los Angeles, California, United States

## Abstract

By 2060, the number of older adults with Alzhemier’s Disease or related dementias (ADRD) is expected to encompass approximately 13.9 million Americans. Recognizing this, the federal government has prioritized research on ADRD and their effect on the physical, emotional, and psychological well-being of persons with dementia (PWD). Such research is complicated by disease-associated decline in cognitive and functional capacity among PWDs which can impair participant ability to process and communicate information, potentially increasing their exposure to negative research-related experiences and compromising the accuracy and reliability of provided data. Nevertheless, as a key stakeholder group, PWD should have the right to participate in research on ADRD. This session will present a case study of ethical issues that emerged in an NIH-funded study utilizing mixed-methods to examine caregiving and care dyad relationships between PWD and their care partners over an 18-month follow-up period. Ethical issues examined will include: (1) PWD capacity to consent to research at baseline and (2) during follow-up visits, (3) Obtaining accurate and reliable data from persons with mild to moderate dementia, (4) Assessing PWD distress while engaging in data collection processes, and (5) Reporting of negative caregiving and life experiences, such as elder mistreatment. The discussion of these topics and presentation of lessons learned holds promise for improving research methodology in studies involving PWD and enabling researchers to increase the involvement of persons with mild to moderate dementia in research on ADRD.

